# Treatments and regulatory mechanisms of acoustic stimuli on mood disorders and neurological diseases

**DOI:** 10.3389/fnins.2023.1322486

**Published:** 2024-01-05

**Authors:** Yikai Chen, Julianne Sun, Junxian Tao, Tao Sun

**Affiliations:** ^1^Center for Precision Medicine, School of Medicine and School of Biomedical Sciences, Huaqiao University, Xiamen, China; ^2^Xiamen Institute of Technology Attached School, Xiamen, China

**Keywords:** acoustic stimuli, music therapy, brain development, mood disorder, neurological diseases, noise exposure

## Abstract

Acoustic stimuli such as music or ambient noise can significantly affect physiological and psychological health in humans. We here summarize positive effects of music therapy in premature infant distress regulation, performance enhancement, sleep quality control, and treatment of mental disorders. Specifically, music therapy exhibits promising effects on treatment of neurological disorders such as Alzheimer’s disease (AD) and Parkinson’s disease (PD). We also highlight regulatory mechanisms by which auditory intervention affects an organism, encompassing modulation of immune responses, gene expression, neurotransmitter regulation and neural circuitry. As a safe, cost-effective and non-invasive intervention, music therapy offers substantial potential in treating a variety of neurological conditions.

## 1 Introduction

Acoustic stimulus, also known as sound stimulus, is defined as specific sounds or noises that elicit a response from an organism’s auditory system (Oxenham, 2012; Goldsworthy, 2022). For centuries, music or beneficial noise have been applied as a non-pharmaceutical therapeutic intervention to enhance physiological and psychological strength in humans (Chen et al., 2022; Lorek et al., 2023). Acoustic stimulus has been shown to improve one’s attention, memory and cognitive flexibility, to influence psychomotor functioning, and to synchronize motor behaviors through rhythm and beat (Braem and Egner, 2018; Terry et al., 2020; Uddin, 2021; Harlow et al., 2023). Music therapies also can reduce arousal levels, and help people gain benefit in managing sleep disorders (Loewy, 2020; Petrovsky et al., 2020). On the other hand, adverse noise stimuli, i.e., acoustic overload or noise pollution, cause a variety of negative physiological and psychological responses (Tao et al., 2020; Fan et al., 2022), and even lead to diseases, for instance permanent hearing loss, fatigue, cognitive impairment, chronic stress responses, and high risk of cardiovascular disorders (Oxenham, 2013; Zhang et al., 2020; Frenis et al., 2021).

Classical music is often used as a therapeutic acoustic stimulus. For example, Mozart’s Sonata for Two Pianos in D Major and Rachmaninov’s Piano Concerto No. 3 in D Minor are commonly employed in therapeutic environments to mitigate negative sentiments (Blood and Zatorre, 2001; Lin et al., 2011; Ding et al., 2023). As a consequence, college students who listened to Mozart performed better on the Stanford Binet’s test than those attended to relaxed rock music or no sound (McLachlan, 1993; Blood and Zatorre, 2001; Pereira et al., 2011). Beside music, nature sounds (Saadatmand et al., 2013; Franco et al., 2017; Thoma et al., 2018), rhythmic drumming (Pantelyat et al., 2016), and lullabies also are used as therapeutic means (Loewy et al., 2013).

We here discuss the impact of different types of sound stimuli, such as music therapy, on the development of the nervous system and the onset and progression of neurological diseases. We summarize strategies and potential mechanisms for harnessing sound stimuli to improve health and circumvent damaging effects of adverse noise exposure.

## 2 Effects of auditory stimulus on infant brain development and functions

Preterm birth, termed as infants born before 37 weeks of gestation, is one of the leading causes of neonatal morbidity and mortality worldwide (Colaizy et al., 2012). An estimated 10.6% of all global births are preterm, leading to approximately 3.1 million annual infant deaths as a direct consequence (Chawanpaiboon et al., 2019; UNICEF, 2023). Beyond its substantial contribution to mortality, effects of preterm birth can persist throughout the life of some survivors, and lead to neurodevelopmental impairments and abnormal brain maturation such as periventricular leukomalacia, neuronal or axonal disorders, characterized by profound cognitive deficits and motor disabilities (Deng et al., 2008; Ligam et al., 2009; Volpe, 2009; Argyropoulou, 2010; Van'T et al., 2015; Cismaru et al., 2016; Gui et al., 2019).

Notably, preterm infants and children who participated music therapy demonstrated stabilized vital signs, marked reductions in heart and respiratory rates, and heightened oxygen saturation levels (Kobus et al., 2021, 2022). This effect was particularly pronounced among 20 preterm infants, born before 32 weeks of gestation, who engaged in biweekly music therapy such as impromptu personal lullaby performances and the use of a sansula, a wooden ring with an attached small kalimba, by the music therapist (Kobus et al., 2021). A clinical trial also showed that involving 21 out of 40 preterm infants who have received approximately eight-minute musical compositions twice daily via study-specific headphones demonstrate significant benefits (Sa et al., 2023). As revealed by multishell diffusion Magnetic Resonance Imaging studies, cortical paralimbic regions of these infants displayed a significantly higher longitudinal increase in fiber cross-section and orientation dispersion index upon music treatment such as calming backgrounds and melodies from bells, harps, and snake flutes (Sa et al., 2020, 2023). Moreover, amygdala volumes in preterm infants were significantly increased after musical interventions based on diffusion tensor imaging studies (Haslbeck et al., 2020; Sa et al., 2020).

Furthermore, mild differences were observed in fear and anger reactivity between the music-exposed preterm group and the full-term group at 12 and 24-month periods, suggesting a positive influence of music exposure on fear processing and potential long-term benefits (Lejeune et al., 2019; Ormston et al., 2022). A study of 47 nine-month-old infants, who were exposed to metered music such as waltzes, also showed evoke neural responses to temporal irregularities in both music and speech after 12 sessions of 15 min each of corresponding activity over a 4-week period in the laboratory, as confirmed through magnetoencephalography (Zhao and Kuhl, 2016). Moreover, musically trained children and adults displayed advanced abilities in processing musical pitch and meter compared to their untrained counterparts (Zhao and Kuhl, 2016; Nan et al., 2018; Putkinen et al., 2019). These findings suggest that music interventions can improve the ability of infants and adults to recognize and anticipate auditory patterns, skills crucial for understanding both music and speech (Moreno et al., 2009; Zhao and Kuhl, 2016; Zhao et al., 2017; Nan et al., 2018; Putkinen et al., 2019; Bower et al., 2021; Lenc et al., 2023).

## 3 Acoustic stimulus in improving sleep quality and treating mood disorders

**Figure 1:**
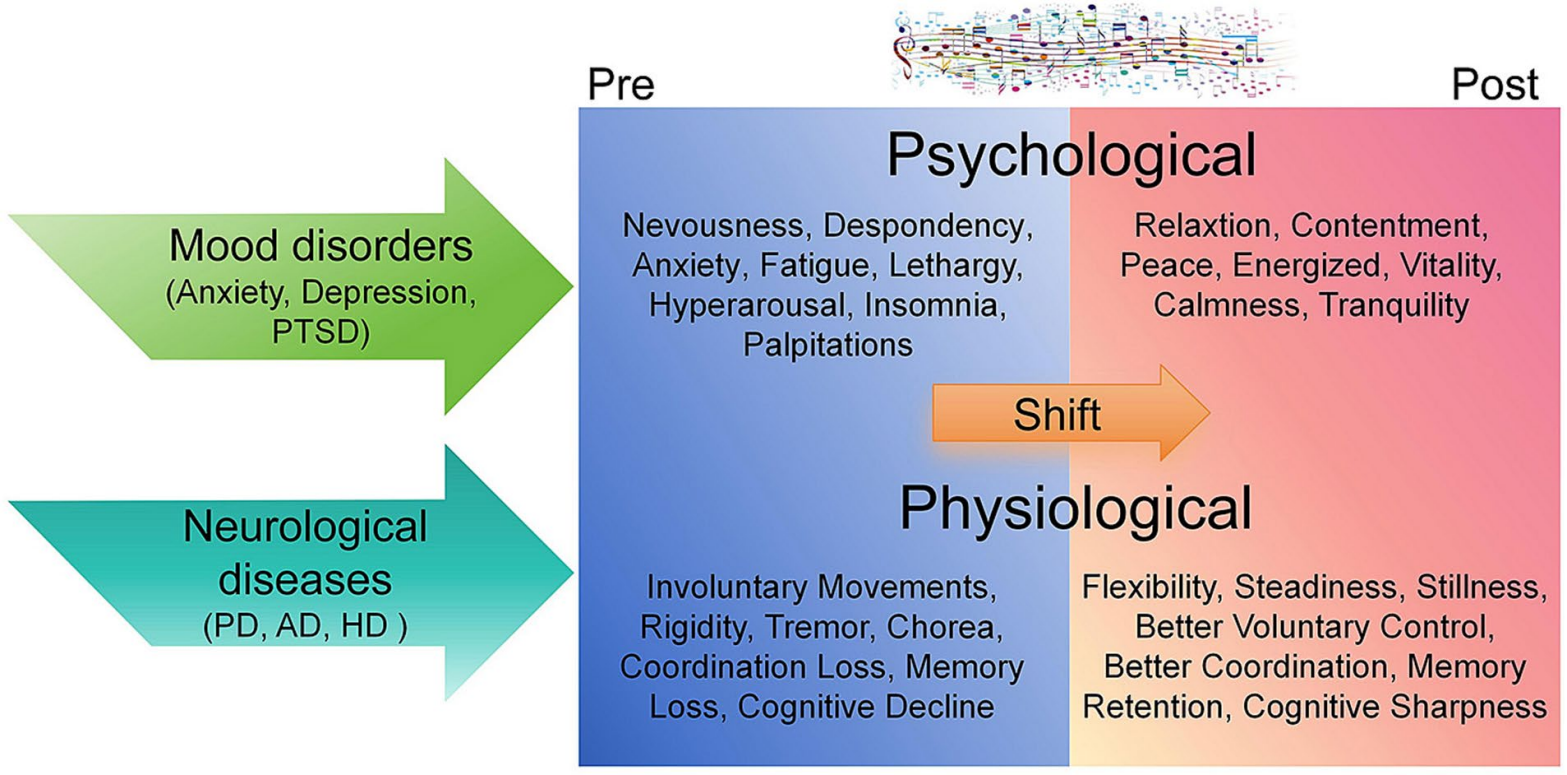
Psychological and physiological shift of pre- and post-music therapy in patients of mood disorders and neurological diseases. The transition from psychological distress and neurological impairments to psychological well-being and physiological stability through music therapy is depicted (Guetin et al., 2009; Ozdemir and Akdemir, 2009; Loewy, 2020; Chen et al., 2021; Matziorinis and Koelsch, 2022; Fan et al., 2023; Navarro et al., 2023). Initial symptoms such as anxiety, tremors, and cognitive decline are characteristic of mood disorders (Fan et al., 2023) and neurological diseases in pre-therapy states (Church et al., 2018; Schwartz et al., 2019; Jones et al., 2020; Matziorinis and Koelsch, 2022; Ghai, 2023). Following therapy, improved relaxation, cognitive acuity, and motor control are evident (Guetin et al., 2009; Ozdemir and Akdemir, 2009; Metzler-Baddeley et al., 2014; Pezzin et al., 2018; Loewy, 2020; Chen et al., 2021; Matziorinis and Koelsch, 2022; Fan et al., 2023; Ghai, 2023; Navarro et al., 2023).

Sleep serves as a critical indicator of an individual’s physical, psychological and social health (Clement-Carbonell et al., 2021). Adequate sleep can regulate emotions, consolidate memory, enhance learning ability, and promote overall well-being (Rasch and Born, 2013; Papalambros et al., 2017; Ramar et al., 2021). Insufficient sleep not only increases the risk of obesity and cardiovascular diseases, but also triggers anxiety, depression and other mental disorders, leading to a decline in cognitive performance (Ward et al., 2014; Muto et al., 2016; Di Muzio et al., 2020; Scott et al., 2021). The Sleep Ambient Music Intervention is an effective therapeutic measure for improving sleep quality and mental health (Loewy, 2020; Chen et al., 2021) (Figure 1). After a four-week music intervention, university students showed improved subjective sleep quality, shortened sleep onset latency, and reduced symptoms of anxiety and depression (Loewy, 2020; Hu et al., 2023). Listening to music during warm-up exercises can significantly enhance the reaction speed, cognitive performance, and average physical power of both individuals suffering from partial sleep deprivation and those with normal sleep patterns (Ballmann, 2021; Khemila et al., 2021) (Figure 1). Their post-exercise cortisol levels and negative emotional states also were effectively reduced (Ballmann, 2021; Khemila et al., 2021; Bentouati et al., 2023).

Attention-Deficit/Hyperactivity Disorder (ADHD) is a prevalent neurodevelopmental disorder primarily affecting children and adolescents, with symptoms often persisting into adulthood (Sayal et al., 2018; Ayano et al., 2023; Park et al., 2023). The primary symptoms of ADHD, including inattention, hyperactivity, and impulsivity, frequently impair individuals’ performance in academic, occupational, and social contexts (Thapar and Cooper, 2016; Cabral et al., 2020; Kautzky et al., 2020; Posner et al., 2020). Interestingly, music therapy has demonstrated effectiveness in treating ADHD (Purper-Ouakil et al., 2011; Meinzer et al., 2016; Shapero et al., 2021). A significant increase in serotonin (5-HT) secretion and a decrease of cortisol levels, blood pressure and heart rate were observed in 18 children and adolescents with ADHD and depression, following music therapy such as spontaneous composition and listening to music with each session lasting 50 min twice a week for 3 months, as compared to the ADHD control group with no treatments (Park et al., 2023). Positive changes in psychological measurements including the Child Depression Inventory and the Depressive Health Questionnaire also were noted (Park et al., 2023). These results underscore promising neurophysiological and psychological benefits of music therapy as an alternative treatment approach for children and adolescents with ADHD, though further research is required to better understand the effect and mechanisms of music therapy for ADHD (Park et al., 2023).

Music has been used to act as a powerful medium for emotional regulation, characterized by alterations in melody and rhythm that match different emotional states (Mitterschiffthaler et al., 2007; Fernandez-Sotos et al., 2016; Feng et al., 2019; Koelsch, 2020) (Figure 1). A significant reduction in depression ratings among 251 children aged 8–16 years old with social, emotional, behavioral and developmental disorders was observed after 12 weeks of playing with musical instruments such as guitars, xylophones and keyboards (Porter et al., 2017). Moreover, 79 adults from 18 to 50 years old with unipolar depression exhibited significant improvements in depression symptoms, anxiety symptoms and general functioning after receiving 20 bi-weekly sessions of music therapy including activities ranging from listening to music to playing, singing songs and free improvisation, compared to those receiving standard psychological interventions (Erkkila et al., 2011). Follow-up assessments further indicated that the positive effect of music therapy can be sustained for at least 6 months (Erkkila et al., 2011).

Post-Traumatic Stress Disorder (PTSD) is a mental health condition often triggered by a life-threatening or traumatic event such as personal assault or a serious accident (Jones et al., 2020). It is characterized by symptoms like flashbacks, nightmares, severe anxiety, and uncontrollable reflection on the incident, all of which significantly degrade the quality of life (Church et al., 2018; Jones et al., 2020). A notable 14.3% decrease in average PTSD severity scores and a 20.4% reduction in depressive symptoms were reported in 40 veterans with moderate to severe PTSD after undertaking 6 weeks of personalized 1-h individual guitar coaching sessions (Pezzin et al., 2018). The improvement in symptoms through active participation in music may not only be ascribed to the music itself but could also be associated with other factors, such as elevating self-esteem through learning new skills, introducing their preferred hobbies, or the impact of personal expression (Pezzin et al., 2018) (Figure 1). Moreover, a randomized controlled trial involving 13 volunteers of 8 males and 5 females with a mean age of 45.7 years old demonstrated a significant improvement in both objective and subjective sleep efficiency and a significant reduction in depression levels following music relaxation in mitigating insomnia among individuals with PTSD (Blanaru et al., 2012). These findings suggest that pre-sleep music relaxation can be used as a therapeutic intervention for treating insomnia in patients with PTSD (Blanaru et al., 2012). Current studies remain preliminary, more research is necessary to specify the precise role of music in therapeutic interventions for PTSD (Pezzin et al., 2018).

## 4 Therapeutic potential of acoustic stimulus in treating neurological disorders

Music therapy also has been employed in the treatment of neurodegenerative diseases such as Alzheimer’s disease (AD) and Parkinson’s disease (PD) (Matziorinis and Koelsch, 2022) (Figure 1). AD is characterized by progressive deterioration of both autobiographical and semantic memory, cognitive function, language skills, and alterations in emotion and behaviors (Matziorinis and Koelsch, 2022; Aguree et al., 2023). The impact of music therapy on AD primarily lies in its ability to evoke memories in patients, to improve their mood and to alleviate stress (Cuddy and Duffin, 2005; Fang et al., 2017). Music therapy also can enhance social participation among AD patients, which is critical to prevent further deterioration of the disease (Cuddy et al., 2015; Peck et al., 2016). Despite challenges in recalling significant autobiographical memories, AD patients can robustly recognize familiar melodies (Cuddy and Duffin, 2005; Peck et al., 2016; Shankar, 2022).

Music-evoked autobiographical memories are prompted by musical cues, provoking immediate emotional reactions and spontaneously revealing information about an individual’s past (Cuddy et al., 2015; Shankar, 2022). Brief excerpts of popular music serve as potent triggers for autobiographical memories among healthy individuals (Janata et al., 2007; Matziorinis and Koelsch, 2022). Remarkably, studies have shown that even exposure to unfamiliar music can enhance episodic memory recall in AD patients (Irish et al., 2006; Matziorinis and Koelsch, 2022). Accompanied by music, these patients displayed significant improvements in recalling autobiographical memories and a notable reduction in trait anxiety (Guetin et al., 2009; Ozdemir and Akdemir, 2009; Navarro et al., 2023) (Figure 1).

PD is a neurodegenerative disorder characterized by symptoms such as tremors, bradykinesia, limb rigidity and balance issues (Fan et al., 2023). The principal use of music therapy in treating PD has been focused on enhancing patients’ motor skills (Fan et al., 2023). Studies indicated that the rhythm of music serves as an external catalyst, aiding patients in refining their gait and movements, a method known as Rhythmic Auditory Stimulation (Fan et al., 2023) (Figure 1).

Moreover, Huntington’s disease (HD) is the most prevalent autosomal dominant neurodegenerative disorder, presents early and selective pathology in the basal ganglia (Reiner et al., 2011). The disorder is often characterized by psychiatric disturbances and motor deficits, including irritability, depression, anxiety, emotional dysregulation and involuntary choreiform movements (Julien et al., 2007; Molnar et al., 2021; Csehi et al., 2023). A study of five HD patients indicated that drumming exercises of 15 min per day 5 times per week for 2 months considerably improve speech and pronunciation, and reduce choreiform movements (Metzler-Baddeley et al., 2014) (Figure 1). Additionally, Traumatic Brain Injury (TBI) is a brain dysfunction caused by an outside force, usually a violent blow to the head (Ghai, 2023). Studies have shown that music therapy can be used to improve gait, coordination, attention, memory, mood, and social interaction in individuals with TBI (Sihvonen et al., 2022; Ghai, 2023).

Furthermore, as a safe, cost-effective and non-invasive intervention, music can alleviate various types of pain, including those related to cancer, surgical procedures, inflammatory conditions, pediatric lumbar punctures and prostate biopsies (Nguyen et al., 2010; Hole et al., 2015; Lee, 2016; Kyriakides et al., 2018). For example, both receptive music therapy and group music therapy have proven effective in reducing pain, stress, depression, anger and anxiety in breast cancer patients (Lagattolla et al., 2023). Music remains beneficial even when the patient is under general anesthesia, and can improve their post-operative experience (Hole et al., 2015).

## 5 Potential mechanisms of how acoustic stimuli function

### 5.1 Endocrine and immune system

It is still not clear how acoustic stimuli influence physiological and psychological conditions in humans. Emerging evidence has shown that innate and adaptive immunity can be influenced by acoustic stimuli, with effects varying based on duration, character and intensity of the sound (Qin et al., 2012; Cui et al., 2016; Zhang et al., 2020). Short-term or low-intensity sound such as the music of Mozart can enhance immune function by decreasing total IgE levels and latex-specific production in peripheral blood mononuclear cells, and skewing the cytokine pattern toward a Th1 type (Kimata, 2003). However, prolonged exposure to noise, such as traffic and mechanical sounds, impairs immune function by decreasing the number and activity of immune cells such as T-lymphocytes, natural killer (NK) cells and phagocytes (Folch et al., 1991; Archana and Namasivayam, 2000; Pascuan et al., 2014; Vasilyeva et al., 2017).

It is likely that neuroendocrine system is responding to acoustic stimuli. Studies indicated that the Hypothalamic-Pituitary-Adrenal axis and the Sympathetic Adrenal Medullary system are regulated by sound exposure, which in turn affects levels of hormones such as adrenaline, noradrenaline, angiotensin II and cortisol in peripheral blood, and regulates expression levels of cytokines like TNF-α, IL1, and IL-17, and eventually the body’s immune responses (Kight and Swaddle, 2011; Pascuan et al., 2014; Hsiao et al., 2015; Recio et al., 2016). It appears that positive sound exposure such as classical music can alleviate stress, promote proper function of the endocrine system and activate the immune system, while inappropriate or excessive noise exposure, for instance long-term traffic and construction noise, can stress the body, disrupt the endocrine system, excess immune responses and lead to occurrence of autoimmune diseases (Rogers et al., 1983; Roswall et al., 2017; Andersen et al., 2018).

### 5.2 Gene expression levels

Accumulating studies suggest that music stimulus promotes brain development and function by elevating levels of Brain Derived Neurotrophic Factor (BDNF), ceruloplasmin, and alpha-1-acid glycoproteins in the brain and serum (Wang et al., 2023). BDNF is a key molecule of neurotrophic factor families and plays a pivotal role in plastic changes of learning and memory (Huang and Reichardt, 2001). After being activated by phosphorylation into p-BDNF, it binds to tropomyosin-related kinase B (TRKB), and promotes receptor dimerization and kinase activation (Fries et al., 2023). Acoustic stimuli can upregulate BDNF expression levels in the hippocampus of adult mice (Chikahisa et al., 2006). Activated BDNF/TRKB pathway enhances downstream signaling pathways such as PLCγ1/PKC, PI3K/AKT, and MAPK/ERK, and in turn promotes neuronal cell survival, proliferation and brain nerve development (Bai et al., 2019; Falcicchia et al., 2020; Li et al., 2021; Wang et al., 2023).

Small noncoding RNAs, in particularly microRNAs (miRNAs) function through regulating target protein coding genes (Bian and Sun, 2011; Zhang et al., 2012; Bian et al., 2013). When individuals were exposed to Mozart’s Violin Concerto No. 3 in G major, expression of several miRNAs were affected, notably an upregulation of miR-23a (Nair et al., 2021). It’s likely that miR-23a enhances long-term neurological function, inhibits neuron apoptosis, and reduces neuroinflammation by inhibiting its target gene PTEN and activating the AKT/mTOR signaling pathway (Lin et al., 2013). Moreover, studies have shown that miR-132 and miR-23b display substantial upregulation in human peripheral blood upon exposure to Mozart’s Violin Concerto No. 3 in G major (Nair et al., 2021; Walgrave et al., 2023). It might in part explain the music effect on treating AD, as studies indicated that miR-132 combats AD by regulating the Tau protein level, preventing its aggregation, alleviating memory deficits, and restoring hippocampal neurogenesis (Liu et al., 2021).

These studies underscore that musical stimuli likely play a significant role in regulating gene expression, and in turn affect brain development and the progression of neurodegenerative diseases.

### 5.3 Neurotransmitters

**Figure 2:**
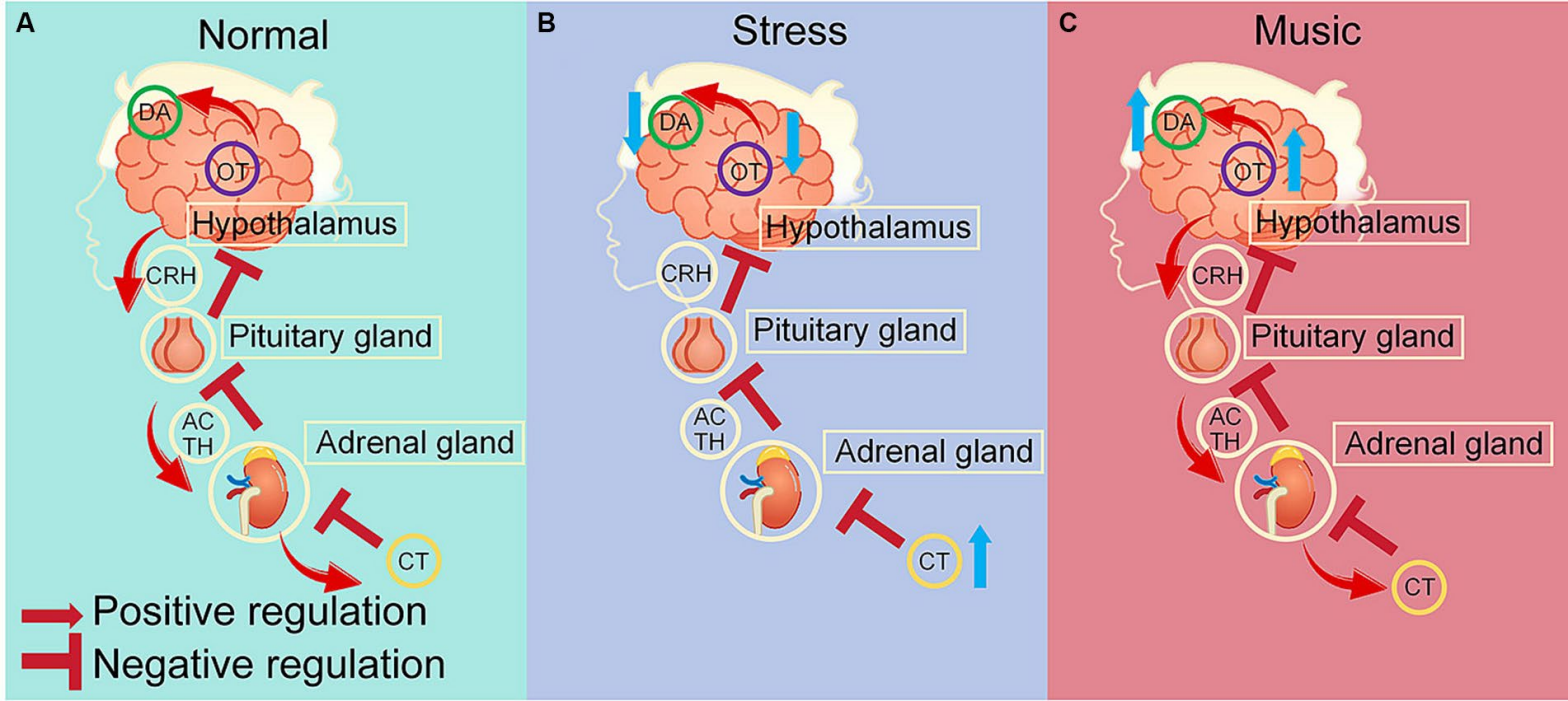
Neurotransmitter regulation by stress and acoustic stimuli. **(A)** Under normal conditions, dopamine, oxytocin maintain a dynamic equilibrium through interactions via the D2R and OTR receptors, oxytocin and cortisol maintain a reciprocal relationship via the hypothalamic-pituitary-adrenal (HPA) axis (Baskerville and Douglas, 2010; Tops et al., 2012; Love, 2014; Alley et al., 2019; Takayanagi and Onaka, 2022). **(B)** Under stress, increased cortisol levels trigger a negative feedback mechanism that downregulates the HPA axis and diminishes oxytocin levels, leading to downregulation of dopamine via the D2R-OTR interaction (Alley et al., 2019; Takayanagi and Onaka, 2022). **(C)** Musical stimuli normalize cortisol levels that are elevated due to stress, thereby restore the dynamic equilibrium of the HPA axis, and upregulate oxytocin, which in turn enhances dopamine levels through D2R-OTR interactions (Baskerville and Douglas, 2010; Tops et al., 2012; Love, 2014; Alley et al., 2019; Rasing et al., 2022; Xie et al., 2022; Okyay and Ucar, 2023). DA, dopamine; OT, oxytocin; CT, cortisol; CRH, corticotropin-releasing hormone; ACTH, adrenocorticotropic hormone.

Dopamine, oxytocin and cortisol are important neurotransmitters, and their complex interplays form a vital neuroendocrine network responsible for modulating various physiological and psychological processes (Baskerville and Douglas, 2010; Tops et al., 2012; Love, 2014) (Figure 2A). The mesocorticolimbic dopamine system, which is crucial for reward processing and expression of affiliative behaviors, consists of interactions between oxytocin and dopamine (DA) that is integral to controlling motor functions, motivation, reward, and social behaviors (Baskerville and Douglas, 2010). This interaction influences various social behaviors and contributes to the pathology of dopamine-dependent disorders such as addiction and mood disorders (Baskerville and Douglas, 2010).

Moreover, oxytocin and cortisol maintain a reciprocal relationship (Alley et al., 2019) (Figures 2A,B). Cortisol, the primary stress hormone, is regulated by the hypothalamic-pituitary-adrenal (HPA) axis, and can be triggered by stress and in turn affect oxytocin levels (Alley et al., 2019). Interestingly, oxytocin may function as a stress buffer and mitigate stress responses by reducing cortisol levels, thereby potentially promoting prosocial behaviors and stress resilience (McQuaid et al., 2016; Young et al., 2021; Takayanagi and Onaka, 2022). Exogenous oxytocin administration has been shown to decrease cortisol levels, reinforcing its potential therapeutic role in stress-related and social disorders (Li et al., 2019). Conversely, cortisol can also modulate oxytocin levels, indicating a bidirectional and complex regulatory mechanism (McQuaid et al., 2016). A study indicated that baseline oxytocin levels are inversely related to cortisol levels, suggesting that higher cortisol might be associated with lower oxytocin (Dumont, 2021). Cortisol administration induced a decrease in oxytocin associated with adrenocorticotropic hormone (ACTH) suppression, and an increase in oxytocin independent of ACTH suppression (Tops et al., 2012). Moreover, under pathological conditions such as PTSD, both oxytocin and cortisol are dysregulated, but their interaction persists even when their levels are abnormal (Li et al., 2019).

In summary, the balance and feedback loops among dopamine, oxytocin and cortisol are critical for emotional stability and the body’s response to stressors (Takayanagi and Onaka, 2022) (Figures 2A,B). The regulatory systems controlling the expression and actions of these neurotransmitters are complex, involving both central and peripheral components of the nervous system (Yuhi et al., 2017). While precise mechanisms of their interactions are still being elucidated, they have significant implications for understanding and treating various conditions involving stress and social behavior dysregulation (Uvnas-Moberg et al., 2015).

Interestingly, studies have shown that music might affect various brain functions through dopaminergic neurotransmission, making it potentially effective in treating symptoms of diseases associated with dopamine dysfunction (Sutoo and Akiyama, 2004). Previous research has shown that calcium boosts DA synthesis via a calmodulin (CaM)-dependent system (Fischer et al., 2020). Elevated DA levels correspond to reduced blood pressure in spontaneously hypertensive rats (SHR) (Figure 2). Exposure to Mozart’s music K.205 reduced systolic blood pressure in SHR, and significantly escalated serum calcium levels and neostriatal DA levels (Sutoo and Akiyama, 2004). These findings suggest that music enhances calcium/CaM-dependent DA synthesis in the brain, leading to a decrease in blood pressure.

Moreover, in HD patients, abnormal dopamine function significantly contributes to the motor and cognitive symptoms of HD (Jamwal and Kumar, 2019). Evidence from positron emission tomography and autoradiography has demonstrated that dopamine signaling in HD is disrupted early on, as indicated by a reduction in striatal dopaminergic D1 and D2 receptor density (Antonini et al., 1996; Weeks et al., 1996; Van Oostrom et al., 2009). Clinical studies have revealed an increase in dopamine levels in early stage HD patients, whereas a decrease in late-stage (Bernheimer et al., 1973; Kish et al., 1987; Garrett and Soares-da-Silva, 1992). It appears that the increase in dopamine synthesis induced by music stimulation helps regulate dopamine levels in HD patients, and has a positive impact in their cognitive, psychological and motor functions (Schwartz et al., 2019; Speranza et al., 2022; Tyagi et al., 2023) (Figure 2C).

Additionally, mood changes post exposure to music or noise seems correlated with the dopamine D2 receptor gene (*DRD2*) gene (Quarto et al., 2017). Studies have shown that people with the *DRD2* rs1076560 GG variant have improved mood and reduced prefrontal activity after music exposure, while those with the *DRD2* GT variant display worsened mood and increased striatal activity after noise (Quarto et al., 2017). Thus, the influence of sounds such as music and noise on individuals’ mood and emotions vary substantially, possibly due to genetic differences for instance the *DRD2* gene (Quarto et al., 2017).

Studies have indicated that activities such as singing (Wulff et al., 2021; Bowling et al., 2022), listening to unfamiliar, sad music (Eerola et al., 2021), and group drumming (Yuhi et al., 2017) can significantly elevate endogenous oxytocin levels, suggesting a positive regulation of oxytocin levels by music stimuli, regardless of forms of the musical activity. It appears that prenatal music and singing interventions can be implemented to enhance expectant mothers’ moods and happiness and to support the mother-infant relationship, since oxytocin levels, expectant mothers’ feelings of happiness and perceived intimacy and closeness to the unborn baby were significantly increased for pregnant women undergoing music listening and singing during pregnancy (Wulff et al., 2021; Dagli and Celik, 2022). Furthermore, embryonic rodent studies also showed effects of oxytocin to reduce stress when exposed to Mozart’s K448 over eight consecutive days (Takano et al., 2022).

Furthermore, cortisol plays a significant role in a variety of mental and neurodegenerative disorders, such as depression, chronic stress, PTSD, fatigue syndromes, dementia and AD (Cheour et al., 2022; Rasing et al., 2022; García-González et al., 2023; Patel et al., 2024). These mental disorders are often profoundly associated with abnormally elevated levels of cortisol, which can severely damage physiological and mental health if maintained over a long period (Patel et al., 2024). Elevated cortisol levels have been observed in 70% of patients with depression and in individuals with AD (Linnemann and Lang, 2020; Rasing et al., 2022). Interestingly, studies have indicated that various forms of music therapy, including improvisational music creation, music listening (Shoda et al., 2023) and group singing (Shoda et al., 2023), can significantly reduce cortisol levels, alleviate anxiety, restlessness, depression, and improve cognitive function and the sense of well-being (Rasing et al., 2022; Xie et al., 2022; Okyay and Ucar, 2023; Shoda et al., 2023) (Figure 2C). Anxiety levels and salivary cortisol levels in women who received music therapy after miscarriage also were significantly reduced (Okyay and Ucar, 2023).

In summary, neurotransmitters play significant roles in brain development, maturation and function (Choi et al., 2023). Restoring neurotransmitter levels is considered one of the effective methods to treat PD and Huntington’s diseases (Jamwal and Kumar, 2019). Accumulating studies have illustrated that music stimuli can effectively regulate neurotransmitter levels, improve brain development, and treat diseases such as AD and PD (Kandimalla and Reddy, 2017; Jamwal and Kumar, 2019; Yang et al., 2023). Moreover, music can significantly increases the levels of oxytocin in the body while significantly reducing cortisol levels (Ooishi et al., 2017; Bowling et al., 2022; Xie et al., 2022). These results indicate that music exerts a significant effect on mental health by balancing levels of oxytocin and cortisol, and by enhancing dopamine levels (Ooishi et al., 2017; Sabino et al., 2020; Bowling et al., 2022; Osorio et al., 2022; Xie et al., 2022). The future research is required to explore mechanisms of music actions by examining changes in the levels of all these different neurotransmitters and their intrinsic relationships.

### 5.4 Neural circuits in response to acoustic stimulus

#### 5.4.1 Frontal striatum circuit

Music’s profound effect on the frontal striatum circuit, which encompasses dopamine pathways interlinking the striatum with various areas of the prefrontal cortex, is underscored by its ability to modulate both psychological and physiological markers of pleasure and emotional arousal (Haber, 2016; Mas-Herrero et al., 2018). Studies employing transcranial magnetic stimulation revealed that this circuit’s activation markedly increases perceived pleasure and emotional arousal during music listening, providing causal evidence of its role in esthetic appreciation and reward (Mas-Herrero et al., 2018). Additionally, interactions of the frontal cortices with the basal ganglia during musical experiences support involvement of the striatal pathway in the temporal and structural processing of sound patterns (Tanaka and Kirino, 2016). Listening to pleasurable music prompts dopamine release within this circuit, further highlighting its integral role in the reward system and in the perception and valuation of musical stimuli (Mas-Herrero et al., 2018; Simpson et al., 2022).

#### 5.4.2 Empathy circuit

Empathy, a cornerstone of social interaction, involves mirroring the sensory and emotional experiences of others, a process intimately connected with music (Koelsch et al., 2021). Studies have demonstrated that empathic traits influence neural activity within the anterior insula and anterior cingulate cortex, key regions implicated in empathy which during music listening (Wallmark et al., 2018). Particularly, individuals with high levels of empathy exhibit intensified activation in these areas when enjoying familiar tunes, indicating a heightened pleasure response (Medford and Critchley, 2010; Taruffi et al., 2021). The anterior insula, recognized as a central hub for empathetic processing, is particularly responsive to the emotive qualities of music, highlighting its role in fostering an emotional resonance between the music and listener (Mutschler et al., 2013). Furthermore, the anterior cingulate cortex, with its ties to both emotional and cognitive facets of empathy, is also actively engaged when individuals are moved by music (Medford and Critchley, 2010). This nexus of brain activity not only elucidates the neural underpinnings of empathy but also reveals how music can be a potent catalyst for emotional understanding and empathic connections (Medford and Critchley, 2010; Laneri et al., 2017).

#### 5.4.3 Corticothalamic circuit

The interplay between auditory stimuli and pain perception is mediated by the corticothalamic (CT) circuits, with the auditory cortex (AC), thalamic posterior nucleus (PO), and ventral posterior nucleus (VP) playing central roles (Zhou et al., 2022). Research has demonstrated that certain sounds, including white noise at a specific sound pressure level above ambient noise, can significantly induce analgesia in mice (Zhou et al., 2022). This effect is facilitated by the CT pathways, which orchestrate the integration and distribution of sensory information (Brunelle and Llano, 2023). The analgesic effect of sound is dependent on a low signal-to-noise ratio, indicating a precise auditory modulation is required to trigger these pathways (Zhou et al., 2022). Furthermore, the auditory cortex is functionally connected to regions involved in nociception, indicating a broader network at play. Studies have shown that even without direct acoustic stimulation, the selective activation of these CT circuits can replicate the analgesic effects of sound (Zhou et al., 2022). This underscores the potential for therapeutic applications of music and sound in pain management, leveraging the nuanced understanding of the role of CT circuits in pain processing (Kuner and Kuner, 2021, 2022).

Noticeably, both human and rodent models have been used to study effects of music stimuli. Mouse and human brains exhibit strikingly similar inhibitory circuit motifs, particularly within the cerebral cortex neural circuits (Wong et al., 2023). This similarity implies a fundamental commonality in their information processing mechanisms (Wong et al., 2023). Orthologous genes between mice and humans also reveal broad patterns of neuroanatomical organization (Beauchamp et al., 2022). However, the human brain is significantly larger and more complex than that of a mouse, featuring a greater number of neocortical folds and specialized regions (Wong et al., 2023). The human cerebral cortex contains a substantially higher neuron count compared to that of the mouse (Loomba, 2022). Thus, even though mouse models are indispensable for neuroscience research due to their resemblance to human brains, substantial differences in brain structure, circuitry, and gene expression require meticulous consideration and methodological improvements.

Taking together, the effect of music stimulus on the body is largely achieved by influencing various neural circuits in the brain such as the frontal striatum circuit, empathy circuit, and corticothalamic circuit. The response of neural circuits to music stimulus can improve the brain’s perception, memory, emotional regulation pain management.

## 6 Perspectives and challenges of acoustic-based interventions

Current studies have shown promising effects of acoustic-based interventions (ABIs) for various health conditions, leveraging the wide engagement of brain circuits by music and sound. Effects of music on regulating movement, emotion, learning and behaviors are likely tied to its ability to engage multiple neural systems (Trimble and Hesdorffer, 2017; Dingle et al., 2021; Chen et al., 2022). The complexity of ABIs poses several challenges such as therapeutic doses, characterization of ABI’s effects across diverse populations, and optimization of intervention strategies (Cuddy et al., 2015; Leuk et al., 2020; Chen et al., 2022; Edwards et al., 2023). Therefore, building on current evidence, a therapeutic music toolbox for brain disorders could encompass customized playlists targeting specific symptoms, rhythm-based exercises for distinct disorders. Such a toolbox can be designed to be evidence-based, widely accessible, and tailored to individual patient needs based on collaborations among neuroscientists, music therapists, and clinicians to ensure its efficacy and applicability.

Scientifically, future research should delve into examining intricate neural mechanisms underpinning therapeutic effects of ABIs, for example, understanding neural circuits involved in rhythm perception and the auditory-motor system by applying brain imaging, brain stimulation and computational analyses (Chen et al., 2022).

## 7 Conclusion

Acoustic stimuli, specifically music, have been demonstrated to have significant effects on various aspects of human health, from the development of preterm infants to adult mental health and neurodegenerative disorders. Music therapy has been shown to provide considerable benefits in stabilizing vital signs, promoting brain maturation, enhancing sleep quality, alleviating symptoms of mood disorders, and managing neurological diseases such as AD and PD. Compelling evidence also suggests that long-term exposure to noisy environments may lead to hearing loss, elevated stress levels, and subsequent adverse health outcomes such as sleep disorders and cognitive decline. Music therapy can improve health conditions by modifying endocrine and immune responses, gene expression levels, as well as the functionality of different brain circuits and pathways. Although many of the mechanisms behind music therapy are still being elucidated, the therapeutic potential of music and other acoustic stimuli is immense. Further research into the regulatory effects of music therapy on the body could help identify specific targets for drug development and medical treatment plans, and explore more effective and viable healthcare strategies to improve public health.

## Author contributions

YC: Writing – original draft, Conceptualization, Investigation, Visualization. JS: Writing – original draft. JT: Writing – original draft. TS: Writing – review & editing.
